# Rabies

**Published:** 1897-11

**Authors:** 


### Rabies.

---

Mr. John P. Haines, the president of the American Society for the Prevention of Cruelty to Animals, thus concludes an article in a recent number of the society's periodical, *Our Animal Friends:*

We have said, and we repeat, that hydrophobia is one of the rarest of diseases; and that when it appears to be developed, we believe it, in the vast majority of cases, to be a simulated disease produced by a morbid imagination. We do not go so far as to assert that it is *never* caused by the bite of a rabid animal; and therefore we would advise that all proper care should be taken to destroy, without delay, all animals that are affected with rabies. Yet, here again, we must recall the fact that rabies itself is one of the rarest of all the diseases with which dogs and other animals are affected. When we hear the cry of "Mad dog!" the chances are millions to one that the dog is not mad; it is the people who are mad with terror. When we read newspaper reports of the appearance of a mad dog here or there, the chances are enormously against the truth of the story. Yet *there is such a disease as rabies;* and since there is no cure for it but death, an animal which is really rabid ought to be killed immediately.

We must remember, however, that the symptoms of rabies are not what they are generally supposed to be. An animal, for example, is not rabid because it avoids water; the rabid dog laps water eagerly. So, a dog is not mad because he froths at the mouth; when he does so, it is a proof that he is not mad. The secretion from the mouth of a really rabid dog is not froth; it is a thick, ropy substance, altogether unlike froth, which the animal tries in vain to expel, at which he sometimes tears with his fore paws, and from which he often seeks relief by drinking. In view of this and other popular mistakes of the symptoms of rabies, our society has put out a leaflet, of which we subjoin a copy.

In another article Mr. Haines says:

What are the Dog Days? They are the "heated term in July and August, during which dogs are supposed to be peculiarly liable to *rabies*, or canine madness." That is one answer, but there is a better.

There are no dog days, because there is no time of the year when dogs are specially liable to rabies. There are no more cases of rabies in July and August than in December and January.

Moreover, rabies is one of the rarest of canine diseases. When you hear a cry of "Mad dog!" in the street, the chances are many thousands to one that *the dog is not mad.* When you read in the newspapers that some one has been bitten by a mad dog, the chances are thousands to one that *it is not true.*

If a human being is bitten by a mad dog, is he not doomed to die a fearful death by hydrophobia? Not at all; for hydrophobia in a human being is much more rare than rabies in a dog. Expert physicians who have given special attention to the subject are convinced that hydrophobia is never caused by the bite of a dog, and that it is simply a hysterical nervous disease caused by an unfounded dread. *Don't take that for granted;* but remember these facts: *first*, that there are more than a million of chances to one that any dog which is supposed to be mad is not mad at all; *second*, that, in all probability, any dog by which a person may happen to be bitten is not mad; and *third*, that even if a person is bitten by a dog that really is mad, the danger of hydrophobia is very slight indeed.

What is to be done if you happen to be bitten by a dog that is supposed to be rabid? The best thing you can do is just to take a few vapor baths, as hot as you can bear them. The perspiration will eliminate any poison that the bite *may* have introduced into your system. Then endeavor to forget all about it. If you follow this simple advice, the chances are incalculably great that you will be perfectly safe.

But is there such a thing as rabies, and such a thing as a mad dog? Undoubtedly there is, though I have never seen one. In the thirty years since the American Society for the Prevention of Cruelty to Animals was established, our officers and agents have been constantly on the outlook, but no undoubted case has ever fallen under their observation, or within their knowledge; and of over a hundred and sixty thousand dogs, and other small animals, which have been cared for at our shelter during the past three years, not one single case of rabies has been found.

These facts sufficiently prove that rabies is rare in this city and in this State; but *there it such a disease*, and it is important for the public, as well as yourself, that you should know whether a sick dog is or is not rabid. If you will note the following *facts*, you will have no difficulty. You will probably find them to be quite different from the popular fancies by which most persons are misled.

1. It is supposed that a mad dog dreads water. It is not so. The mad dog is very likely to plunge his head to the eyes in water, though he can not swallow it and laps it with difficulty.

2. It is supposed that a mad dog runs about with evidences of intense excitement. It is not so. The mad dog *never runs about* in agitation; he never gallops; he is always alone, usually in a strange place, where he jogs along slowly. If he is approached by dog or man, he shows no sign of excitement, but when the dog or man is near enough, he snaps and resumes his solitary trot.

3. If a dog barks, yelps, whines, or growls, that dog is not mad. The only sound a mad dog is ever known to emit is a hoarse howl, and that but seldom. Even blows will not extort an outcry from a mad dog. Therefore, if any dog, under any circumstances, utters any other sound than that of a hoarse howl, that dog is not mad.

4. It is supposed that the mad dog froths at the mouth. It is not so. If a dog's jaws are covered or flecked with white froth, that dog is not mad. The surest of all signs that a dog is mad is a thick and ropy brown mucus clinging to his lips, which he often tries vainly to tear away with his paws or to wash away with water.

5. If your own dog is bitten by any other dog, watch him carefully. If he is infected by rabies, you will discover signs of it possibly in from six to ten days. Then he will be restless, often getting up only to lie down again, changing his position impatiently, turning from side to side, and constantly licking or scratching some particular part of his head, limbs or body. He will be irritable and inclined to dash at other animals, and he will sometimes snap at objects which he imagines to be near him. He will be excessively thirsty, lapping water eagerly and often. Then there will be glandular swellings about his jaws and throat, and he will vainly endeavor to rid himself of a thick, ropy, mucus discharge from his mouth and throat. If he can, he will probably stray away from home and trot slowly and mournfully along the highway or across the country, meddling with neither man nor beast, unless they approach him, and then giving a single snap. The only exception to this behavior occurs in ferocious dogs which, during the earlier stage of excitement, may attack any living object in sight.

These symptoms of rabies are condensed from valuable information received from physicians of undoubted authority—*N. Y. Medical Journal.*

